# Right Sizing: Sensory-Based Product Design Is a Promising Strategy to Nudge Consumers toward Healthier Portions

**DOI:** 10.3390/nu10101544

**Published:** 2018-10-19

**Authors:** David Labbe, Lisa R. Fries, Aurore Ferrage, Francine Lenfant, Nicolas Godinot, Nathalie Martin

**Affiliations:** Nestlé Institute of Material Sciences, Nestlé Research, Vers-chez-les-Blanc, 1000 Lausanne 26, Switzerland; david.labbe@rdls.nestle.com (D.L.); lisa.fries@rdls.nestle.com (L.R.F.); aurore.ferrage@rdls.nestle.com (A.F.); francine.lenfant@rdls.nestle.com (F.L.); nicolas.godinot@alimentarium.org (N.G.)

**Keywords:** sensory, food design, portion size, nudging, behavior, food choice

## Abstract

Research has shown that people consume more food when offered larger portions, and that reducing exposure to large food portions and packages could decrease the average daily energy consumed. In this context, our aim is to develop strategies to promote healthier eating behaviors by reducing portion selection and intake. The present research investigates the impact of different visual attributes of foods on quantity perception and portion selection. In the first study, we tested whether modifying the shape of a familiar food influenced the ideal portion size in adults. In the second study, we assessed the impact of shape, number of units, size, and color variety on a perceived quantity for a familiar multiunit product in children. Participants (*N_1_* = 70 adults, *N_2_* = 62 children) completed different picture-based computer tasks. As hypothesized: (1) adults selected a smaller ideal portion size for an elongated product than for wider and thicker shapes, and (2) children’s perception of food quantity was primarily driven by number of pieces, with smaller effects of size and elongation. Perceived quantity was not influenced by color variety. These findings suggest that it may be possible to reduce the size of food portions without negatively impacting perceived quantity, and to provide opportunities to nudge consumers towards smaller portions while maintaining satisfaction.

## 1. Introduction

Overweight and obesity are caused by many factors, including an increase in portion sizes. These larger portions, combined with an increase of the number of eating occasions, have been identified as key modifiable drivers of intake [1,2]. As explained in a recent Cochrane review by Hollands et al. [3], people select and consume more food when offered larger portions or packages, as well as when using larger tableware items (e.g., plate size). The authors suggest that reducing exposure to large food portions and packages could decrease the average daily energy consumed.

Different factors have been suggested to influence portion size selection and intake [4]. Among sensory factors that are key drivers of the eating experience, visual cues seem to play a significant role in the amount of food selected and eaten [5]. Visual cues that can influence perception of quantities, portion selection, and intake include the shape, number, and size of food units, as well as the color variety. These factors and their associations with perception of quantity and portion size for foods and beverages are described in greater detail in the sections that follow.

### 1.1. Elongation and Size

There has been evidence of the impact of elongation on perceived quantity since Piaget’s studies in the 1960s about how children learn about conservation of mass and volume [6,7]. Piaget observed that children tended to perceive taller, narrower containers to hold more liquid than short wide ones, and hypothesized that this illusory distortion was due to the child’s attention being centered, or focused, on the vertical dimension, which therefore would account for the relative overestimation of the size of taller containers. Similar biases in the perception of liquid volumes have been demonstrated in adults. Raghubir and Krishna [8] showed that elongation increased the perceived volume of drink containers or glasses. Wansink and van Ittersum [9] also reported that the volume poured by adults (and children) was larger in a short wide glass than in a tall slender glass. The same (albeit attenuated) effect was observed in professional bartenders [9]. Finally, one study also showed that making one dimension more salient by adding lines on the object could modulate the perception of its size [10].

Although the role of container shape and size on portion selection and consumption has been widely studied, the impact of the shape of the food itself has been underexplored [11]. In children, the impact of food shape has been explored with respect to food acceptance [12,13,14], but the impact of shape on portion selection or intake has not been tested. To our knowledge, only a few studies have explored the impact of food shapes in adult portion selection and intake. One found that cutting food (carrots and surimi gel) into fine strips increased perceived quantities compared to blocks or small cubes in adults [15].

### 1.2. Item Number and Size in Multi-Item Servings 

The reduction in portion selection when food is presented in smaller pieces has been reported in adults for a variety of foods, including biscuits [16,17], candies [18], pretzels [19], and pizza slices [20]. In adults, this “segmentation heuristic” has been proposed to explain that, when presented with small pieces, people eat more units but do not fully compensate for the unit-size decrease [17]. It has been suggested that multiple pieces of food are more rewarding to both humans and animals because they are perceived as larger quantities since they take up a larger surface area [11]. The impact of presenting food in multiple smaller units on expected satiety has also been recently demonstrated [21]. It is also possible that adults may consume less of foods segmented into smaller units because they perceive eating so many pieces to be a sign of overindulgence [20] or impulsivity [22]. There has been much less recent attention to this segmentation heuristic in children. However, Marchiori and colleagues [23] showed that cutting cookies in half can result in children reducing their intake by 25%.

### 1.3. Color Variety

Variety in different sensory attributes of food (appearance, flavor, texture) influences food intake through the “sensory specific satiety” phenomenon in humans, i.e., the decline of pleasantness for the taste of an eaten food compared to an uneaten food [24,25]. When different sensory properties are varied at the same time (e.g., color, texture, flavor), people eat significantly more of the food [26]. However, results are mixed when considering the influence of color only. In a study exploring the impact of color variety on children’s consumption of chocolates, there was no difference in intake between children in the single-color or color-variety conditions [25]. In another study, increasing the variety of a set of colored candies only increased portion selection when the candies were organized and divided into individual bins by color, but not when the colors were mixed together [27]. However, when adult participants in another study were asked to pour candies in a bowl to match a reference candy bowl, the adults pouring multicolored candies poured significantly more than those pouring candies of a single color.

### 1.4. Hypotheses Tested in the Current Study

The first aim of our study was to investigate the effect of elongation of foods on food-related decisions to complement the existing literature, which primarily focuses on the shape of containers rather than the food itself. We tested the impact of elongation of two different products (ice cream and candies) for two different populations (adults and children). In Study 1, we hypothesized that adults would select a smaller portion for an elongated ice cream than for an ice cream with a standard or wider shape because of the increased size impression of the elongated shape. We also tested, as a secondary outcome, whether making one dimension more salient through the addition of horizontal or vertical lines would modulate this elongation effect. In Study 2, our hypothesis was that elongating a familiar product (bear-shaped gummy candies) would increase the amount perceived by children compared to the same number of candies with a standard shape, even when holding the volume constant.

The second aim of our work was to confirm the role of the number of units rather than their size in multiunit serving on the evaluation of the overall food amount. In study 2, we aimed to confirm the segmentation effect reported in children in a previous study [23] by dissociating the effects of size and number, to demonstrate that children’s perception of quantities in a multi-item serving is driven by number, independently of the size of each unit. Indeed, in previous studies, unit size and number of units have varied concurrently and it is difficult to disentangle the two potential effects: larger units have systematically been presented in reduced numbers compared to smaller units in order to hold the overall quantity presented as constant. From a young age, children can differentiate small quantities of items by number, whereas estimating the area or volume of an object is a more challenging mental calculation even for adults, requiring the ability to consider multiple perceptual dimensions and make an overall judgment. For this reason, we expected that the children in our study may have defaulted to simpler heuristics to solve the problem of creating fair piles. Therefore, we expect that number, as an early developing skill, may drive fairness judgments for most children.

Finally, the third aim of our research was to investigate the role of color in the perception of quantity in children in a novel experimental context using a smaller countable number of food items. As the previous literature has been mixed on the effect of color variety on portion selection, we did not have any strong a priori hypotheses on this variable and included it as an exploratory analysis.

Our different hypotheses were tested using picture-based computer tasks. The “method of adjustment” [28] that we had successfully applied in previous work to quantify ideal portion size [29] was used in Study 1 in adults. For Study 2, we chose and adapted a different task to be suitable to the cognitive ability of children from six to eight years old, and to avoid the expectation that they would receive large quantities of candies at the end of the experiment. This task is based on the “Matched Fullness” task [30] using a projective scenario to assess perceived quantity of gummy candies in children.

## 2. Study 1

### 2.1. Methods

#### 2.1.1. Participants

Seventy American women in the range of 35–55 years old who were consumers of ice cream and declared having consumed chocolate-coated vanilla ice cream sticks at least 6 times in the past 6 months were recruited in San Diego (United States) for a central location test. Participants did not have any known food allergy, intolerance, or any food restrictions due to personal beliefs.

The study protocol complied with the criteria of the Market Research Society (MRS) code of conduct. The MRS code of conduct ensures that the privacy rights of human subjects are always observed, and that participants are provided with sufficient information to allow informed consent to be signed before participating in the study. Participants were compensated for their time.

#### 2.1.2. Ice Cream Pictures and Method of Adjustment Task

Six ice cream sticks were designed using CAD software (Solidworks Pro 2014, Dassault Systemes, SolidWorks Corporation, Waltham, MA, USA): a reference shape (REFERENCE), TALLER, WIDER, THICKER shapes varied in length, width, and thickness to modify the shape, while a volume of 90 mL was kept constant across shapes (Table 1). Two additional shapes were replications of the reference shape, with the addition of vertical (REFERENCE V) or horizontal (REFERENCE H) lines on the coating to enhance the salience of the vertical/horizontal dimension and hence potentially modulate the size impression.

For each design, 41 picture variants were created, ranging in size from 50 to 130 mL, with a 2 mL incremental step between each image, while maintaining the same relative proportions. The ice cream stick was presented at an angle so that participants could see the product’s thickness (Table 1).

**Figure 1:**
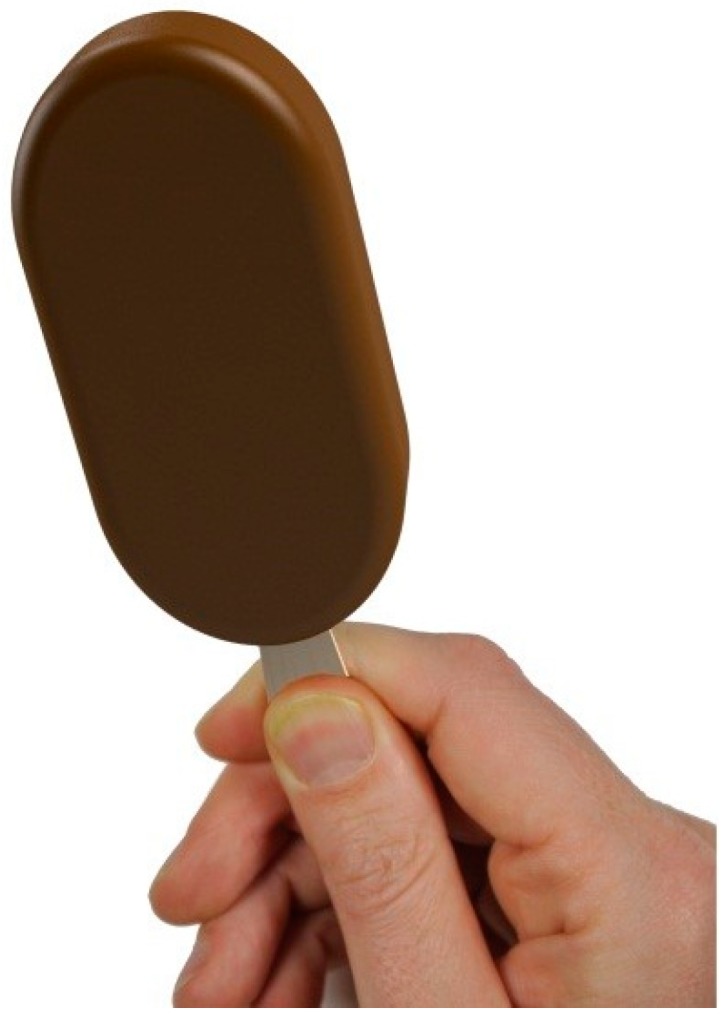
Example of picture used in the adjustment task with ice cream stick displayed on a computer screen in ¾ frontal view.

The “method of adjustment” (Brunstrom and Rogers, 2009) was used to identify self-selected ice cream stick size for each shape. A computer task using E-Prime 2.0 software (Psychology Software Tools, Pittsburgh, PA, USA) was developed to display the ice cream held by the wooden stick in the center of the screen (Figure 1). The hand in the picture provided a frame of reference for the participants’ perception of the product size. The first picture shown on a computer screen was a random image between 70 and 120 mL, and participants were asked to “Imagine it is snack time, choose the bar size that would satisfy you the most” by increasing or decreasing the size of the bar using the up and down keyboard arrows. Participants were instructed that “By satisfying, we mean how well you expect this product to fulfil your needs at snack time”. The six ice cream stick variants were presented to all participants in a randomized order. The task lasted approximately 10 min.

#### 2.1.3. Statistical Analysis

The outcome measure of the task was the volume (mL) of the ideal self-selected portion size for each ice cream shape. The impact of shape on portion size was measured by one-way within-participant analysis of variance (ANOVA) with the “shape” factor set as fixed. Posthoc paired comparisons were assessed by Fisher’s Least Significant Difference (LSD) Test. The confidence level was set to 5% for all statistical analyses, and analyses were conducted using IBM^®^ SPSS^®^ software version 21 (IBM Corporation, Armonk, NY, USA).

### 2.2. Results

**Figure 2:**
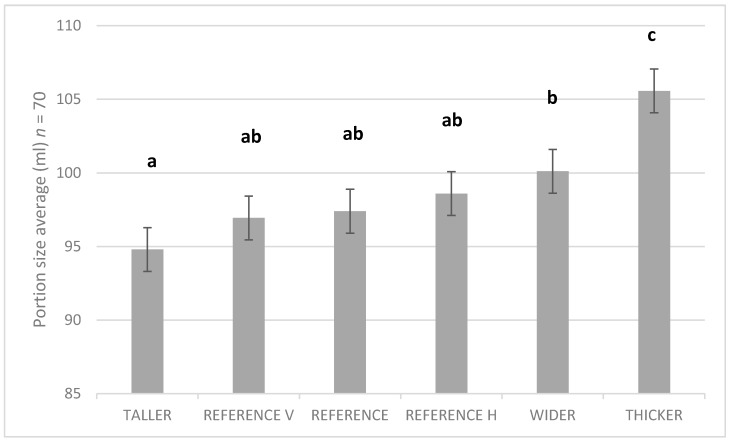
Mean (± SEM) ideal self-selected portion size for the six ice cream shapes. Different letters (a, b, c) account for significant difference between ice cream shapes.

Ideal self-selected portion size for ice cream was significantly impacted by shape (F(5,345) = 11.8, *p* < 0.001) (Figure 2). The smallest selected portion size was observed with the elongated shape, TALLER (mean = 94.8 mL, *SD* = 21.8), which was significantly smaller than the portion size obtained with the WIDER (mean = 100.1 mL, *SD* = 20.4) and THICKER variants (mean = 105.6 mL, *SD* = 21.4). There was a trend for TALLER to be selected in a smaller portion than the REFERENCE shape, although the difference did not reach significance. There was no significant difference between the REFERENCE shape and the shapes with vertical or horizontal lines added on the coating.

## 3. Study 2

### 3.1. Methods

#### 3.1.1. Participants

Children aged six to eight years old and a parent were recruited at three locations in England: Bourneville, Ware, and Staines, through door-to-door recruitment and by telephone from an existing consumer database. All children were reported to like eating and have eaten gummy candies in the past three months. Children used a computer at least once a month. Only one child per household was eligible to participate in the test, they should not have had any known food allergy or intolerance, or any food restrictions due to personal beliefs. Overall, 72 children were initially recruited, 24 per location. Six children participated in pilot sessions and their data were not included in the current analyses. Four children withdrew over the course of the study and their data have been excluded. Data from 62 children (32 girls) with a mean age of 7.6 years (*SD* = 0.27) were included in the analyses. Written informed consent was obtained from all parents and the assent from children was verbally collected at the beginning of the study. Families were compensated for their participation and children received a small bag of sweets. The protocol was submitted to the UK Health Research Authority (HRA) to enter the ethical review process. On the basis of the application and confirmation that the research participants would be healthy volunteers, the HRA concluded that the study fell into the framework of a market research study, sourcing volunteers from a market research database and therefore a review by the Research Ethics Committee within the HRA was not required. The study was then carried out in accordance with the MRS code of conduct.

#### 3.1.2. Stimuli: Matching Task and Photographs

The objective of the task was to understand the impact of number, shape, size and color variety on quantity perception through children’s selection of “fair” portions of candies.

Bear-shaped gummy candies were selected as a reference product and five other prototypes were produced, of which the first two were modified in shape through elongation or flattening, keeping their depth (9 mm) and weight constant (3.5 g). The other three variants that were created were reduced in size by 30% by proportionally reducing both the height and width of the three previously mentioned variants (Table 2). Each of the six gummy variants was presented in four different numbers: three, five, eight, and 12. These quantities were selected because they were within the range of typical consumption for this type of product. Only red gummy candies were used to study the impact of shape and size on perceived quantity. A 3 (shape) × 2 (size) × 4 (number) experimental design was built with 24 trials.

The impact of color diversity on quantity perception was investigated through a second experimental design with the REFERENCE gummy shape presented according to a 3 × 3 experimental design with three number levels (five, eight, and 12) and three color varieties (red; red/green; red/green/orange/yellow). The red, green, orange, and yellow color variety was selected since it represented the typical color diversity in commercial gummy bears, and the red and green color combination to enhance color salience, as these two colors showed a strong visual contrast. Six additional trials with the color varieties were added to the size and shape trials, resulting in a total of 30 trials in the matching task. All children completed all trials.

#### 3.1.3. Matching Task

The matching task was developed using E-Prime 2.0 software (Psychology Software Tools, Pittsburgh, PA, USA). We avoided asking the children to select their own ideal portion size as was done in adults, as children may overindulge themselves when allowed to select their own portion of a well-liked product. Instead, we focused on factors driving children’s perception of quantity through a matching game. The matching task was presented as a computer game in which the child was asked to help a cartoon character make two piles of candies for himself and his friend so that both of them can have a fair share. The literature shows that children understand concepts of fairness from a young age, and that school-aged children are generally motivated to fairly distribute resources [31]. By projecting their responses onto cartoon characters, rather than dividing the products between themselves and the experimenter, the task avoided both potential “greedy” responses, and the risk that the child expected to take the candy home.

**Figure 3:**
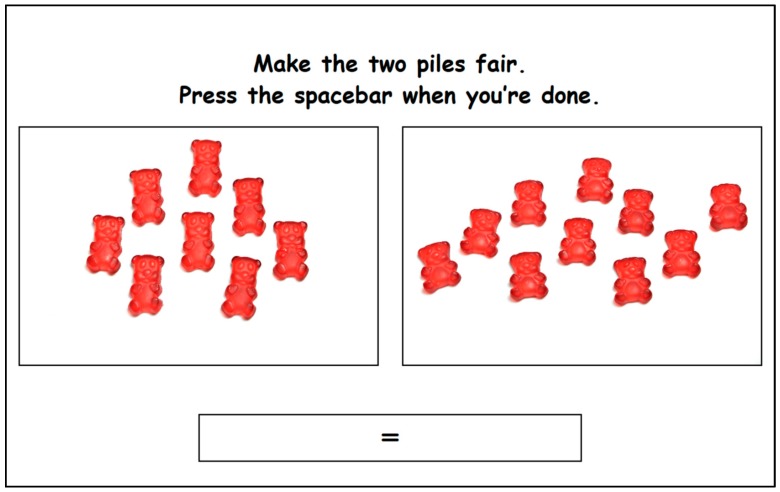
Screen capture of the matching task in which eight TALLER gummy candies are presented on the left (“test”) pile, and 10 REFERENCE gummy candies were selected by the child on the right pile. The sign ‘=’ was used to remind the children to make two fair piles of candies.

Each of the 30 test “piles” were displayed on a piece of paper and photographed with a high-resolution digital camera. The setup for the photography kept the lighting, viewing angle, and camera focus constant across photos. These 30 photographs were presented as the left (“test”) pile, following a randomized order automatically generated by the software. For each test pile, participants were instructed to add between 1 to 24 red REFERENCE gummy candies to the “selection” pile on the right to make the two piles fair. Each of the 24 incremental images of standard gummy candies were photographed following the same protocol described above. Participants selected the appropriate number of reference gummy candies by pushing the up arrow to add a candy to the pile, or the down arrow to take one away. When the piles were perceived to be fair, participants were instructed to press the spacebar to display the next trial (Figure 3). To facilitate the understanding of the notion of fairness and to familiarize the children with the task, training trials were done before starting the real trials with the guidance of the moderator. Orange gummy candies were used for the training trials to illustrate that different numbers (two and seven reference candies), sizes and shapes (an extra-large gummy candy of 29 mm height and 19 mm width) could be presented on the left part of the screen. For the trial with the large bear on the test pile, the experimenter explained that the candies on the left would sometimes be of different shapes and sizes, so the cartoon character would need more help to make the two piles fair. The experimenter continued to explain that if the candy on the left is bigger, the cartoon character might need more small bears to make it fair. For all trials, the child was told that, at the end of the task, one of the cartoon characters would always choose first and would choose the biggest portion (left or right), leaving the other pile for the second character. Therefore, it was important that the children made the piles fair so that both characters would get a fair portion.

#### 3.1.4. Statistical Analysis

We measured the influence of the number, shape, and size of the gummy candies presented on the number of gummy candies selected with a fixed-factor 4 (unit number) × 3 (shape) × 2 (size) ANOVA. The influence of color variety on the number of selected gummy candies was independently investigated through a separate fixed-factor 3 (color variety) × 3 (unit number) ANOVA. Interactions between variables of both ANOVAs were also explored. Partial Eta Squared $\left(\eta_{p}^{2}\right)$ was calculated as a measure of effect size with 0.01, 0.06, and 0.14 as indicative values to consider the effect to be “small”, “moderate”, or “large”, respectively [32]. Posthoc paired comparisons were assessed using Fisher’s LSD tests.

### 3.2. Results

#### 3.2.1. Impact of Size, Shape, and Number

**Figure 4:**
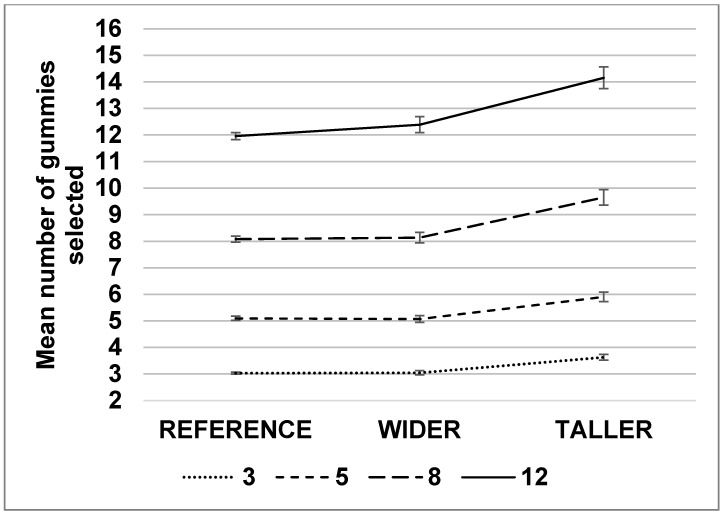
Mean number of gummy candies selected ± SEM for each number of gummy candies presented (3, 5, 8, 12) and shape of candy (REFERENCE, WIDER, TALLER).

The number of presented pieces had a significant effect on the number selected (F(3,1464) = 1302.1; *p* < 0.01, $\eta_{p}^{2}=0.72$). Overall, children selected a number of reference gummy candies close to the number presented in the test pile (Figure 4).

**Figure 5:**
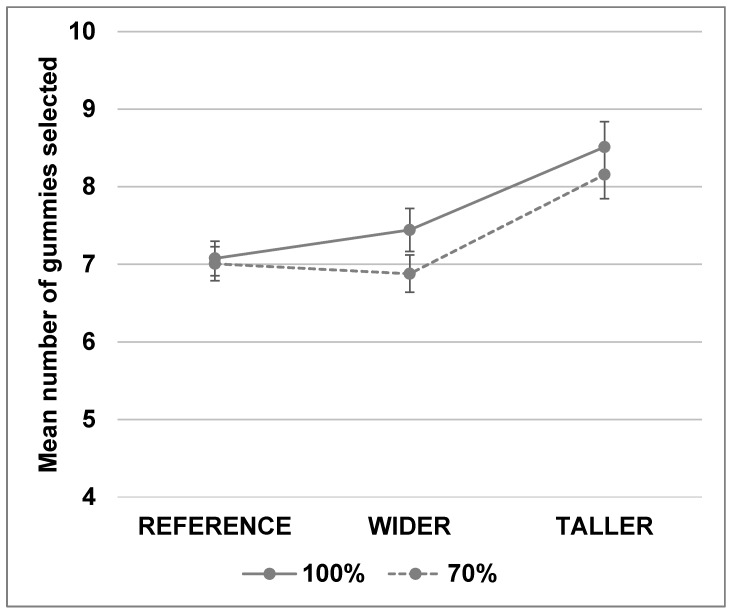
Mean number of gummy candies selected ± SEM for the reference, WIDER and TALLER shape and the two sizes: 100%, 70% (reduced size).

A main effect of the shape on the selected number was also observed (F(2,1464) = 50.6, *p* < 0.01, $\eta_{p}^{2}=0.07$). Overall, across the four number conditions, significantly more pieces were selected to match the TALLER shape (mean = 8.33, SEM ± 0.10) than the WIDER (mean = 7.16, SEM ± 0.10) or REFERENCE shapes (mean = 7.04, SEM ± 0.10) (Figure 5).

There was also a significant main effect of size (F(1,1464) = 8.1, *p* < 0.01, $\eta_{p}^{2}=0.01$) with fewer gummy candies selected when the reduced-size gummy candies were presented (mean = 7.33, SEM ± 0.08) than for the full-sized ones (mean = 7.64, SEM ± 0.08) (Figure 5).

There was a significant interaction between the number of presented gummy candies and the shape (F(6,1464) = 3.7, *p* < 0.01, $\eta_{p}^{2}=0.02$), with slightly greater impact of elongation when the number of presented gummy candies increased (Figure 4). Other interactions between the number of gummy candies and the size (F(3,1464) = 1.07, *p* = 0.35), between the size and the shape (F(2,1464) = 1.54, *p* = 0.21) and between the size, shape, and number (F(6,1464) = 0.38, *p* = 0.89) did not reach significance.

Overall, the number of units showed the strongest effect on perceived quantity, followed by the shape, and then by the size, with partial Eta squared values of 0.72, 0.07, and 0.01, respectively.

#### 3.2.2. Impact of Color Variety

There was no significant effect of the color variety on the number of selected gummy candies (F(2,549) = 1.18, *p* = 0.31, $\eta_{p}^{2}=0.004$). Regardless of the color variety, the number of selected gummy candies was the same as the presented number. In this ANOVA, the number of presented gummy candies was the key driver of the number of gummy candies selected (F(2,549) = 3066.5, *p* < 0.01, $\eta_{p}^{2}=0.92$), as previously observed.

## 4. Discussion

In both studies, we validated our hypothesis about the impact of elongation on portion selection, in line with previous research demonstrating that an elongation visual-distortion bias leads to increased size impression [8,9,33]. In the first study, we observed a difference of 11% in volume between the ideal portions of the two shapes with the most extreme differences (TALLER and THICKER). Product developers aiming to reduce the energy content of a product while maintaining consumer satisfaction need to be conscious of the potential impact of product shape, and they may be able to further leverage the geometric features of products to achieve this goal. Contrary to our expectations, vertical and horizontal lines added on the coating did not significantly influence portion selection. The dark brown lines on a brown coating may not have been salient enough to enhance the elongation effect.

Of note, the thickest shape, THICKER, resulted in a larger selected portion size than the WIDER shape, which was the same height. Elongation (height/width) was larger for the THICKER shape (2.17) than for the WIDER shape (1.62). Considering elongation alone, the THICKER shape should have led to a smaller ideal portion size than the WIDER shape according to Raghubir and Krishna (1999). However, making the third dimension (thickness) in the THICKER shape bigger and thus potentially more salient may have led the participants to underestimate the total volume of this ice cream even more (and subsequently select the largest ideal portion) in line with the effects of three-dimensional change on volume estimation demonstrated by Chandon and Ordabayeva [33]. Another explanation may be that the participants failed to notice differences in thickness due to the more frontal view used to display the different ice cream shapes on the screen. The other dimensions (length and width) may have been easier to see, leading the participants to perceive the THICKER ice cream to be smaller and subsequently to select larger ideal portion sizes.

In the second study, we observed that the number of units had the largest impact on perceived quantity. Then, to a lesser extent, we also showed the effect of elongation on children’s decision processes, in line with Wansink and van Ittersum [9], who reported that children poured and consumed significantly more juice (74% more) when given a short, wide glass compared to those given a tall slender glass. The effect was not as large in our study with solid foods, but elongation still led to a significant increase of 18% in the perceived amount of gummy candies. The number of units was the main driver of perceived quantity in a multi-item context for children, as hypothesized. The significant interaction between number and shape revealed that, when more gummy candies were presented, the effect of elongation was stronger, suggesting that multi-item servings are even more relevant food targets to leverage elongation perceptual bias to reduce portion sizes. This interaction has not been previously documented in portion-size research. One possible explanation could be due to differences in how small quantities and large ones are cognitively processed. Very small quantities (under five) can be subitized, meaning that that the quantity can be estimated automatically without counting. For larger quantities, individuals either need to consciously count them or rely on other cues to make an estimate. In the current study, the shape of the product may be influencing this estimation as children reach larger numbers of pieces.

Unit size had a significant impact on portion selection. Indeed, when children were presented with a specific number of smaller gummy candies (five, eight, or 12), they selected about the same number of standard gummy candies (five, eight, and 12, respectively) to make two fair piles of candies, resulting in an overall higher caloric content in the pile created by the child with standard gummy candies. For instance, when five reduced-size elongated gummy candies (12.5 g, 43 Kcal) were presented, children selected on average 5.8 standard gummy candies as being a fair equivalent (20.3 g, 70 kcal). In Figure 5, the overall unit-size effect seems to be due to the TALLER and WIDER shapes, although the size × shape interaction was not significant. Reducing unit size did not influence children’s quantity perception and subsequent portion selection using standard shapes. It is possible that children were more sensitive to changes in product size in both the TALLER and WIDER conditions, as these unusual shapes were more salient and increased their attention to product dimensions. In contrast, the variant with a standard shape but reduced in size looked “normal” to the children and they may not have paid as much attention to it.

Color variety did not seem to influence perceived quantity. It is possible that there was less impact of the visual effects of color variety compared to previous studies [34], as in the current study, the multicolored gummy candies and red gummy candies were directly compared, whereas in previous studies, samples were not presented simultaneously. Finally, in contrast to the previous studies, the quantity of gummy candies used in our study was still within the range of what is countable, whereas a bowl of M&M’s**®** [34] is beyond that, and becomes an uncountable mass. As previously mentioned, small and large quantities are perceived differently from a cognitive perspective.

Finally, this was the first time, to our knowledge, that a fairness-based computer matching task has been used to assess children’s perception of quantity. As the findings are in line with our hypotheses based on previous work using real foods and containers, this suggests that this method may be a promising alternative way of testing new food concepts with children before producing prototypes.

## 5. Limitations and Future Directions

We demonstrated, using computer tasks, that elongation and unit downsizing make it possible to reduce the size of a portion without negatively impacting perceived quantity for two product categories. Such findings need to be replicated on different types of products to be able to conclude that the effect is generalizable.

Although we hypothesized that the elongation effect was due to an increased size impression of the elongated shape, we did not explicitly test this through a mediation analysis. This could be considered in future studies by asking the participants to report on their perceived amount of food in the different experimental conditions.

Further, future studies need to explore whether responses on these computer tasks reflect actual portion selection and intake. Indeed, very few studies have, to our knowledge, validated that portion selection resulting from such a computer task matches actual portion selection, and results in the literature have been mixed. For example, one study found that computer-based assessments of ideal portion size and expected satiety were good predictors of self-selected portion and food intake [35], whereas another showed that expected satiation measurements based on picture assessment did not correspond to actual consumption [36]. Even if there seems to be a strong relationship between portion selection and intake [37], it is important to measure not only how much people select but also how much they eat to ensure that the solutions developed actually impact intake. It is also possible that the way the computer experiments were designed may have enhanced some of the observed effects. For example, presenting the gummy candies vertically on the computer screen might have enhanced the salience of the elongation effect. In a real consumption context, where the candies are manipulated and can fall in other orientations, the elongation effect may be attenuated.

Finally, the sustainability of the impact of right sizing solutions leveraging different perceptual and cognitive biases (number, unit size, elongation effects) also needs to be carefully assessed because disconfirmation of expectations, habituation, and compensation can occur over the long term. Indeed, some studies have reported short-term discrepancies between expectations made during the portioning stage, consumption, and postconsumption perceptions. For example, one study reported that participants who served themselves smaller volumes in taller, elongated glasses could drink larger volumes from these glasses because their higher expectations about perceived volume in elongated glasses were disconfirmed after drinking. Experiencing less beverage than expected led to dissatisfaction and higher postconsumption [8]. More generally, it is also important to validate the sustainable impact of such nudging strategies in the long term and their potential role in calorie reduction over time because compensation can offset short-term effects. A few studies have investigated the impact of reducing portion size of meals or snacks over a few days [38,39] or months [40], and showed that people do not seem to fully compensate when offered smaller portions, resulting in a reduced overall caloric intake. However, potential compensation mechanisms resulting from offering products modified in their shape, unit number, and size need to be evaluated.

## 6. Conclusions

To conclude, our study shows that it seems to be possible to reduce the size of food portions without negatively impacting perceived quantity through optimization of the food’s visual properties and especially the elongation and the number and size of the elements in multiunit servings. Our findings provide opportunities to direct food design that can nudge consumers towards healthier diets while maintaining satisfaction. These types of changes to the food environment to implicitly guide people to make better choices complement more traditional approaches relying on education (e.g., nutrition knowledge), cognitive control, and willpower (dieting strategies) that may not be sufficient to change behaviors.

## Figures and Tables

**Table 1:** Ice cream stick dimensions and pictures for a constant volume of 90 mL.

Shape	Height; Width; Thickness (mm)	Picture
REFERENCE	94.0; 46.5; 24.5	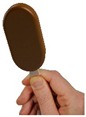
TALLER	105.3; 41.0; 24.5	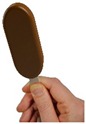
THICKER	89.0; 41.0; 29.5	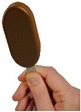
WIDER	89.0; 55.0; 22.0	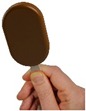
REFERENCE V	94; 46.5; 24.5 with vertical lines	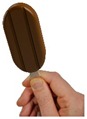
REFERENCE H	94; 46.5; 24.5 with horizontal lines	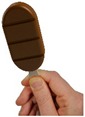

**Table 2:** Reference and variant gummy candy dimensions, weight and pictures.

Shape	Size	Height; Width (mm)	Weight (g)	Picture
REFERENCE	Reference (100%)	25; 19	3.5	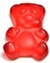
TALLER	Reference (100%)	30; 16	3.5	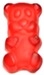
WIDER	Reference (100%)	21; 23	3.5	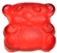
REFERENCE-	Reduced (70%)	21; 16	2.5	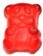
TALLER	Reduced (70%)	25; 13	2.5	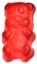
WIDER	Reduced (70%)	17; 19	2.5	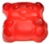

## References

[1] Duffey K.J., Popkin B.M. (2011). Energy density, portion size, and eating occasions: Contributions to increased energy intake in the United States, 1977–2006. PLoS Med..

[2] Young L.R., Nestle M. (2012). Reducing portion sizes to prevent obesity: A call to action. Am. J. Prev. Med..

[3] Hollands G.J., Shemilt I., Marteau T.M., Jebb S.A., Lewis H.B., Wei Y., Higgins J.P.T., Ogilvie D. (2015). Portion, package or tableware size for changing selection and consumption of food, alcohol and tobacco: A cochrane systematic review. Cochrane Database Syst. Rev..

[4] Benton D. (2015). Portion size: What we know and what we need to know. Crit. Rev. Food. Sci. Nutr..

[5] English L., Lasschuijt M., Keller K.L. (2015). Mechanisms of the portion size effect. What is known and where do we go from here?. Appetite.

[6] Miller P.H. (2002). Piaget’s cognitive-stage theory and the neo-piagetians. Theories of Developmental Psychology.

[7] Crain W. (2005). Piaget’s cognitive-developmental theory. Theories of Development: Concepts and Applications.

[8] Raghubir P., Krishna A. (1999). Vital dimensions in volume perception: Can the eye fool the stomach?. J. Mark. Res..

[9] Wansink B., Van Ittersum K.V. (2003). Bottoms up! The influence of elongation on pouring and consumption volume. J. Consum. Res..

[10] Krider R.E., Raghudir P., Krishna A. (2001). Pizzas: Π or square? Psychological biases in area comparisons. Mark. Sci..

[11] Wadhera D., Capaldi-Phillips E.D. (2014). A review of visual cues associated with food on food acceptance and consumption. Eat. Behav..

[12] Branen L., Fletcher J., Hilbert L. (2002). Snack consumption and waste by preschool children served “cute” versus regular snacks. J. Nutr. Educ. Behav..

[13] Boyer L.E., Laurentz S., McCabe G.P., Kranz S. (2012). Shape of snack foods does not predict snack intake in a sample of preschoolers: A cross-over study. Int. J. Behav. Nutr. Phys. Act..

[14] Van Kleef E., Vrijhof M., Polet I.A., Vingerhoeds M.H., de Wijk R.A. (2014). Nudging children towards whole wheat bread: A field experiment on the influence of fun bread roll shape on breakfast consumption. BMC Public Health.

[15] Wada Y., Tsuzuki D., Kobayashi N., Hayakawa F., Kohyama K. (2007). Visual illusion in mass estimation of cut food. Appetite.

[16] Kerameas K., Vartanian L.R., Herman C.P., Polivy J. (2015). The effect of portion size and unit size on food intake: Unit bias or segmentation effect?. Health Psychol..

[17] Weijzen P.L.G., Liem D.G., Zandstra E.H., de Graaf C. (2008). Sensory specific satiety and intake: The difference between nibble- and bar-size snacks. Appetite.

[18] Marchiori D., Waroquier L., Klein O. (2011). Smaller food item sizes of snack foods influence reduced portions and caloric intake in young adults. J. Am. Diet. Assoc..

[19] Geier A.B., Rozin P., Doros G. (2006). Unit bias. A new heuristic that helps explain the effect of portion size on food intake. Psychol. Sci..

[20] Davis B., Payne C.R., Bui M. (2016). Making small food units seem regular: How larger table size reduces calories to be consumed. J. Assoc. Cons. Res..

[21] Oldham-Cooper R.E., Wilkinson L.L., Hardman C.A., Rogers P.J., Brunstrom J.M. (2017). Presenting a food in multiple smaller units increases expected satiety. Appetite.

[22] Van Kleef E., Kavvouris C., van Trijp H.C. (2014). The unit size effect of indulgent food: How eating smaller sized items signals impulsivity and makes consumers eat less. Psychol. Health.

[23] Marchiori D., Waroquier L., Klein O. (2012). “Split them!” Smaller item sizes of cookies lead to a decrease in energy intake in children. J. Nutr. Educ. Behav..

[24] Rolls B.J., Rowe E.A., Rolls E.T. (1982). How flavour and appearance affect human feeding. Proc. Nutr. Soc..

[25] Rolls B.J., Rowe E.A., Rolls E.T. (1982). How sensory properties of foods affect human feeding behavior. Physiol. Behav..

[26] Rolls B.J., Rowe E.A., Rolls E.T., Kingston B., Megson A., Gunary R. (1981). Variety in a meal enhances food intake in man. Physiol. Behav..

[27] Kahn B.E., Wansink B. (2004). The influence of assortment structure on perceived variety and consumption quantities. J. Consum. Res..

[28] Brunstrom J.M., Shakeshaft N.G. (2009). Measuring affective (liking) and non-affective (expected satiety) determinants of portion size and food reward. Appetite.

[29] Labbe D., Rytz A., Godinot N., Ferrage A., Martin N. (2017). Is portion size selection associated with expected satiation, perceived healthfulness or expected tastiness? A case study on pizza using a photograph-based computer task. Appetite.

[30] Brunstrom J.M., Rogers P.J. (2009). How many calories are on our plate? Expected fullness, not liking, determines meal-size selection. Obesity.

[31] Blake P.R., McAuliffe K., Warneken F. (2014). The developmental origins of fairness: The knowledge-behavior gap. Trends Cogn. Sci..

[32] Cohen J. (1988). Statistical Power Analysis for the Behavioral Sciences.

[33] Chandon P., Ordabayeva N. (2009). Supersize in one dimension, downsize in three dimensions: Effects of spatial dimensionality on size perceptions and preferences. J. Mark. Res..

[34] Redden J.P., Hoch S.J. (2009). The presence of variety reduces perceived quantity. J Consum. Res..

[35] Wilkinson L.L., Hinton E.C., Fay S.H., Ferriday D., Rogers P.J., Brunstrom J.M. (2012). Computer-based assessments of expected satiety predict behavioural measures of portion-size selection and food intake. Appetite.

[36] Guillocheau E., Davidenko O., Marsset-Baglieri A., Darcel N., Gaudichon C., Tomé D., Fromentin G. (2018). Expected satiation is correlated with amplitude of intake but does not fit well with actual consumption of desserts. Appetite.

[37] Wansink B., Johnson K.A. (2015). The clean plate club: About 92% of self-served food is eaten. Int. J. Obes..

[38] Rolls B.J., Roe L.S., Meengs J.S. (2006). Reductions in portion size and energy density of foods are additive and lead to sustained decreases in energy intake. Am. J. Clin. Nutr..

[39] Stroebele N., Ogden L.G., Hill J.O. (2009). Do calorie-controlled portion sizes of snacks reduce energy intake?. Appetite.

[40] French S.A., Mitchell N.R., Wolfson J., Harnack L.J., Jeffery R.W., Gerlach A.F., Blundell J.E., Pentel P.R. (2014). Portion size effects on weight gain in a free living setting. Obesity.

